# Primary mantle-cell lymphoma of small intestine presenting with intussusceptions: A case report and review of the literature

**DOI:** 10.1016/j.ijscr.2024.109963

**Published:** 2024-06-27

**Authors:** Mohamed Amine Tormane, Ghazi Laamiri, Hazem Beji, Houda Gazzeh, Mahdi Bouassida, Hassen Touinsi

**Affiliations:** Department of General Surgery, Hospital Mohamed Taher Maamouri, Nabeul, Tunisia; University Tunis El Manar, Faculty of Medicine of Tunis, Tunisia

**Keywords:** Mantle-cell lymphoma, Small intestine, Resection, Case report, Literature review

## Abstract

**Introduction and importance:**

Mantle cell lymphoma is a rare type of non-Hodgkin's lymphoma which accounts for 5 % of all cases.

Patients present with an advanced form of the disease. We present here a case of ileocolic intussusception secondary to mantle cell lymphoma which was revealed by abdominal pain and vomiting that was treated by surgical resection followed by chemotherapy.

**Case presentation:**

This report illustrates the case of a 34-year-old male who presented with abdominal pain and vomiting. Imageology demonstrated an ileocolic intussusception which was treated with hemicolectomy followed by chemotherapy. Histopathology confirmed the diagnosis of Mantle cell lymphoma.

**Clinical discussion:**

Mantel cell lymphoma is a rare type of B-cell cancer. Patients are generally diagnosed with an advanced stage of the disease. Ileocolic intussusception is an uncommon presentation. Surgery is the pillar of the treatment. Resection depends on the extent and location of the lesion. Postoperative chemotherapy is crucial and it increases survival rate.

**Conclusion:**

Mantle cell lymphoma is a rare subgroup of B-cell lymphomas. Ileocolic intussusception is a complicated form of the disease. Surgery combined with chemotherapy is the mainstay of the treatment. Diagnosis is confirmed by histological analysis of the surgical specimen.

## Highlights

• Mantle cell lymphoma is a very rare tumor which belongs to the non-hodgkin lymphoma subgroup.
• Ileocolic intussusception is an uncommon presentation.
• Surgery followed by chemotherapy are the pillar of the treatment.
• Resection depends on the extent and location of the lesion.

## 1 Introduction and importance

Mantle cell lymphoma is a rare subgroup of the broad category of B-cell lymphomas (1). It is a particular type of non-Hodgkin's lymphoma, accounting for around 5 % of all cases (2,3). It is more common in male patients and usually occurs in adults (4). Patients typically present with an advanced stage of the disease which frequently involves the gastrointestinal tract (4). The lower gastrointestinal tract is the most commonly affected part. (5) Bowel intussusception is an uncommon occurrence in adults, with ileocolic localization representing only 13 % of cases (6,7).

Herein, we present the case of a 34-year-old man with ileocolic intussusception secondary to mantle cell lymphoma, which was successfully treated with surgical resection followed by chemotherapy.

This work has been reported in line with the SCARE 2020 criteria (8).

## 2 Presentation of a case

A 34-year-old male complained of acute cramping right-sided abdominal pain, vomiting and bloating for two days.

Furthermore, the patient reports a history of constipation and a milder abdominal pain along with a decline in overall health over the past eight months.

On admission, he was hemodynamically stable; He has a normal blood pressure of 12/7 and he has no tachycardia (pulse at 80 bpm). Abdominal examination revealed a diffuse mild tenderness and distension with and excessive bowel sounds. There was no palpable abdominal mass on physical examination. Blood tests showed a normal white blood cell count (5900 cells/mm3) with a reversed cell type (neutrophils: 68.1 %, lymphocytes: 17.4 %), a moderate decrease in haemoglobin (12.7 g/dl) and a normal platelet count of 152,000 cells/mm3 (Fig. 1a). Biochemistry was within normal range, except for a high CRP level (40 mg/l).

**Fig. 1a:**
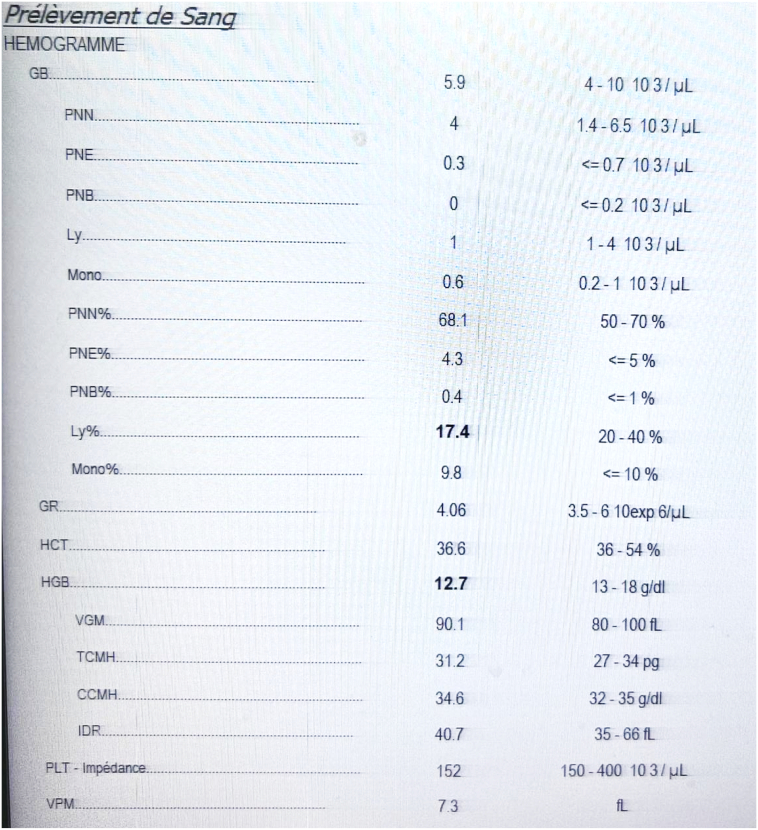
Blood test.

The abdominal CT scan with contrast injection and without opacification revealed the presence of a target sign in the ascending colon, which is suggestive of an ileocolic intussusception upstream of a mass on the wall of the invaginated ileum (Fig. 1b, Fig. 1c).

**Fig. 1b:**
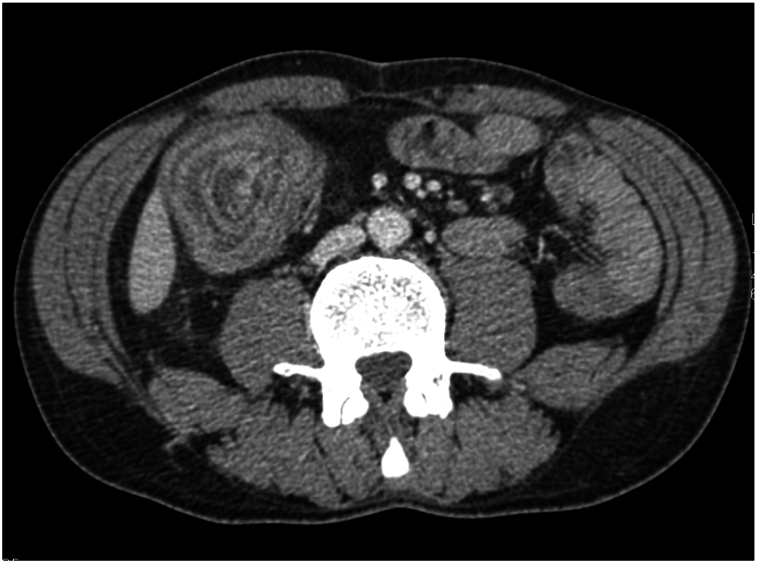
Abdominal CT Scan in the axial plane revealing a target sign in the ascending colon suggestive of an ileocolic intussusception.

**Fig. 1c:**
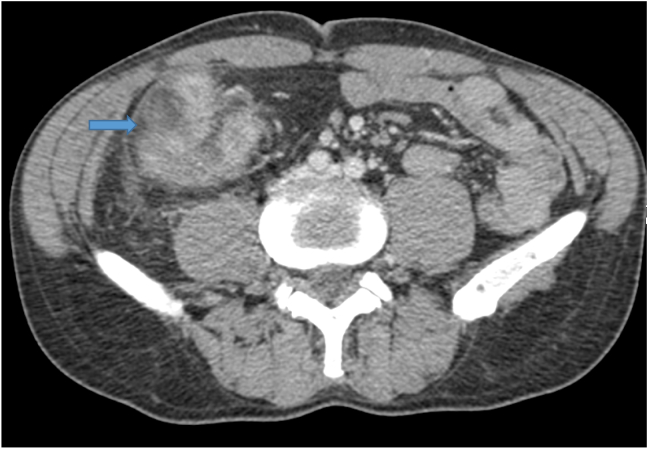
Abdominal CT Scan in the axial plane revealing the intestinal mass.

**Fig. 2:**
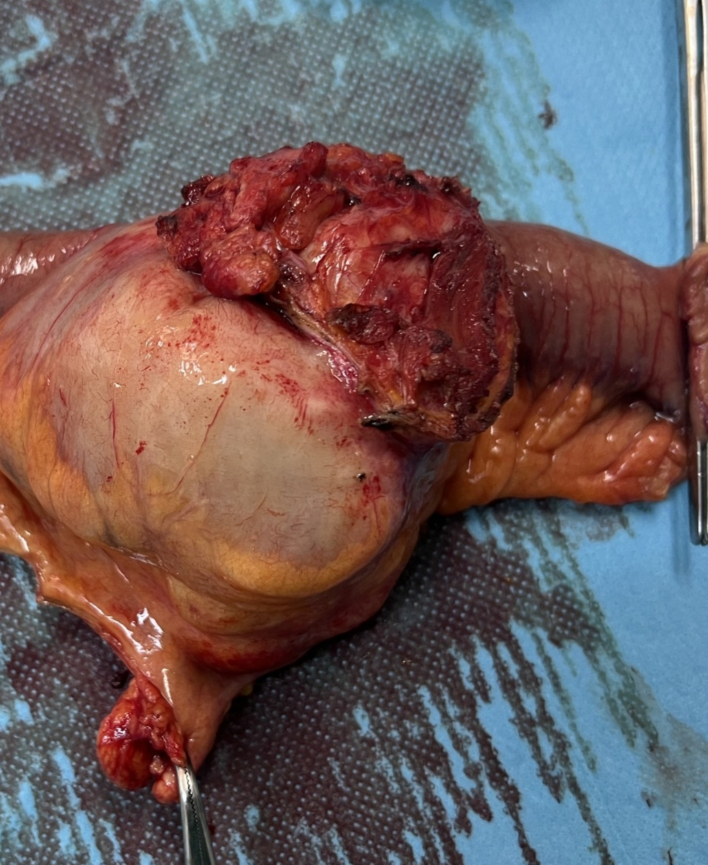
the specimen.

We opted for emergency laparotomy. We found the invagination of the terminal ileum into the ascending colon. A polypoid lesion was found on the wall of the invaginated ileum. We performed a right hemicolectomy removing 15 cm of the distal ileum, the ascending colon and up to mid transverse colon with a hand-sewn side to side ileocolic anastomosis. The specimen was sent for histological analysis (Fig. 2). Operating time was 2 h, there was no blood loss, and the patient was admitted in a low care ward. The postoperative course was uneventful with immediate mobilisation and normal intestinal functionality. He was discharged after 5 days.

Tumor marker CA 125 and CA19–9 were performed after surgery and were within normal range.

A chest-abdomen computed tomography was performed one month after the operation. It did not show the presence of adenopathy or pulmonary metastases.

**Fig. 3:**
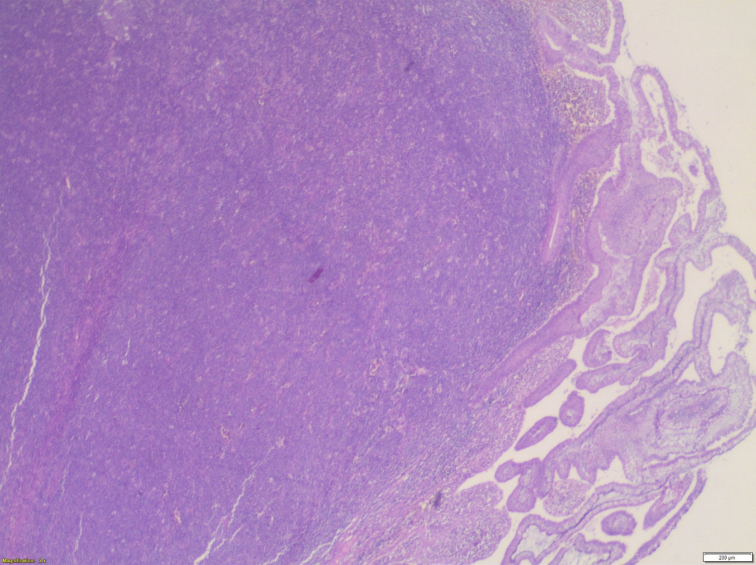
Histopathological slide demonstrating lymphomatous polyposis of the terminal ileum with mantle cell lymphoma.

**Fig. 4:**
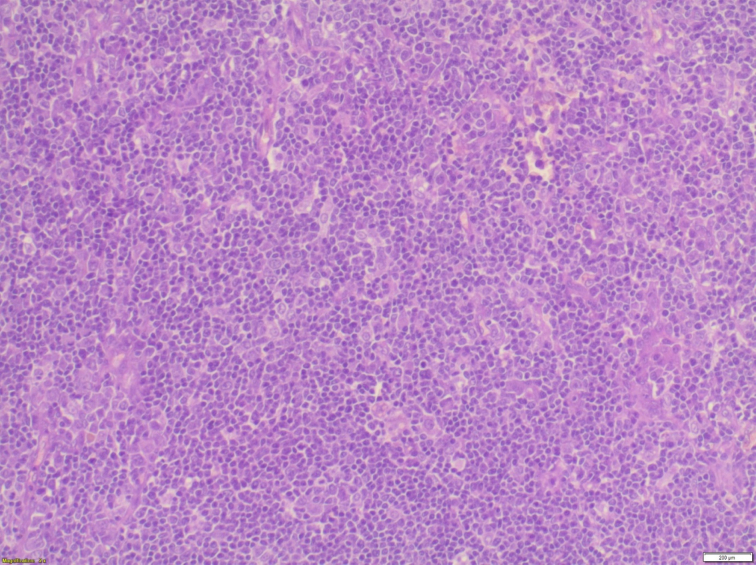
Histopathological slide enlarged 200 times under the microscope(x200) with hematoxylin and eosin (HE) showing fragments of colonic mucosa showing infiltration by atypical lymphoid cells.

**Fig. 5:**
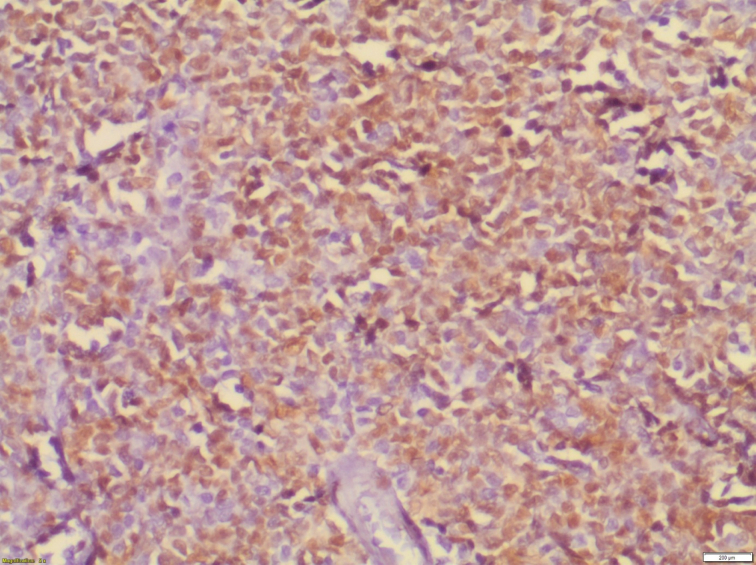
Immuno-histochemistry showing positive Cyclin D1 marker.

**Fig. 6:**
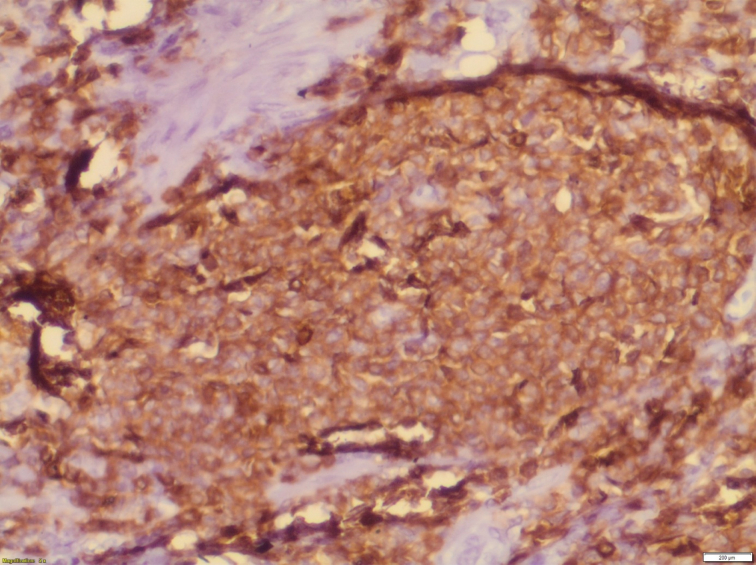
Immuno-histochemistry showing positive CD5 marker.

**Fig. 7:**
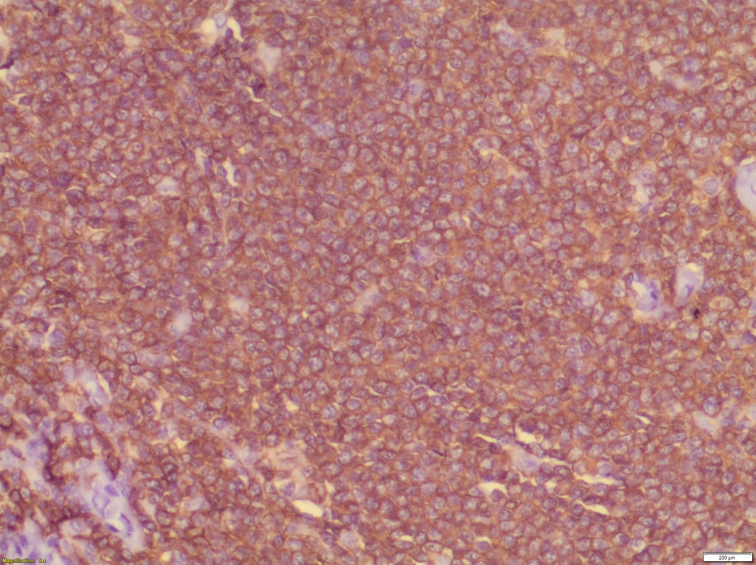
Immuno-histochemistry showing positive CD20 marker.

**Fig. 8:**
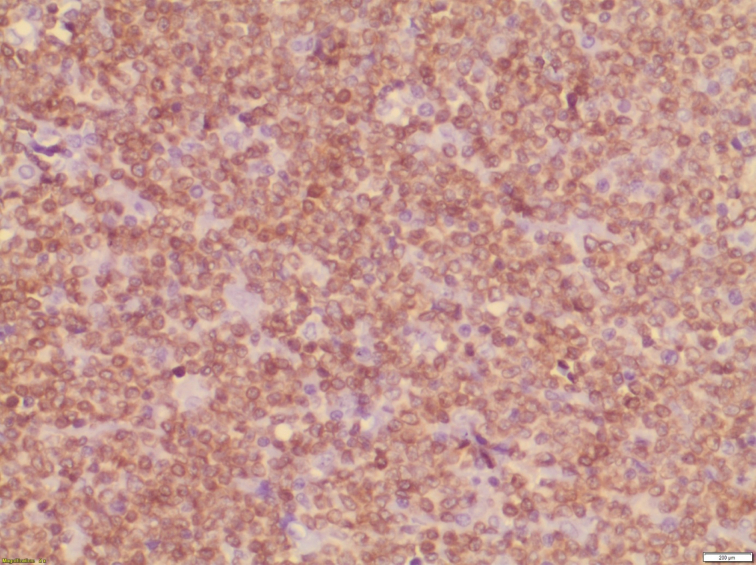
Immuno-histochemistry showing positive BCL2 marker.

Histopathological examination of the specimen revealed lymphomatous polyposis of the terminal ileum with mantle cell lymphoma (Fig. 3) and fragments of colonic mucosa showing infiltration by atypical lymphoid cells **(**Fig. 4). Immuno-phenotypic and Immuno-histochemistry analyses were positive for Cyclin D1 (Fig. 5), CD5 (Fig. 6), CD20 (Fig. 7) and BCL2 (Fig. 8), but negative for CD10, CD23 and BCL6, which confirms which confirmed the diagnosis of stage IV mantle cell lymphoma with intact bowel resection margins.

He underwent 8 cycles of chemotherapy involving rituximab-CHOP (cyclophosphamide, hydroxyadriamycin, vincristine, and prednisone).

He remained in remission with good overall performance after 6 months of follow-up.

## 3 Clinical discussion

We reported a successful surgical resection of ileocolic intussusception secondary to mantle cell lymphoma. The main strength of our work is the timely diagnosis and the performance of the surgical treatment and chemotherapy.

Lymphomas are defined as solid tumors originating in the immune system (9). Hodgkin's lymphoma constitutes approximately 10 % of all lymphomas, while the remaining 90 % are classified as non-Hodgkin lymphoma (1,8).

Non-Hodgkin's lymphomas represent a wide spectrum of malignant tumors affecting the lymphoid system (9,10). These affections have been classified into B-cell and T-cell neoplasms. B-cell lymphomas account for around 90 % of all lymphomas, the most common histological types are follicular lymphoma and diffuse large B-cell lymphoma (11). Mantel cell lymphoma is a type of B-cell cancer which represents approximately 3–6 % of all adult non-Hodgkin lymphomas (1,4). On average, patients are diagnosed at the age of 60, and 75 % of them have advanced disease (stage IV) with generalized lymphadenopathy (11,12). Malignant lymphoma can be found in all systemic organs. Several studies have found that this disease was present in the upper part of gastro intestinal tract in 43 % of patients and in the lower part of the small intestine in 88 % of them (5,13,14). Rarely, it is located in the spleen (15), skin and bone marrow (8,9) making the diagnosis difficult. Mantle cell lymphoma can manifest with symptoms such as recurrent abdominal pain, gastrointestinal bleeding or diarrhea (13). In rare cases, it causes an acute abdomen pain due to intestinal obstruction, Intussusception or perforation (1,12,16).

Intussusception consists of the telescoping of a proximal segment of the intestine into a distal segment (6). It is uncommon in adults and accounts for only 1 % of cases of intestinal obstruction (17).

Upper gastrointestinal endoscopy and colonoscopy are essential means of diagnosing Mantle cell lymphoma, as they can be used to locate polyps and to take tissue biopsies (4). Abdominal CT scan contributes to the positive diagnosis of lymphoma by revealing a mass or polyps in the bowel or colon. It can also be used to diagnose complicated forms. The “sausage shape and target sign on abdominal scan are highly evocative of intussusception (18). Imaging studies must be performed in order to stage the disease, and include evaluation by whole-body CT or PET scan (19). In our case, the patient had a timely abdominal CT scan to confirm the diagnosis of intussusception.

Mantle cell lymphoma cannot be distinguished from adenomatous or hamartomatous polyposis, or from MALT lymphoma, by only endoscopic or radiological evaluation. Histological tissue analysis of the specimen is crucial for a definitive diagnosis (4,20). Histomorphologic and immunophenotypic analysis correspond to mantle cell lymphoma must be positive for Cyclin D1, CD5, CD20 and CD79a and negative for CD10, CD23 and BCL16 (19,21). In our case, this tests were also positive for Cyclin D1, CD5 and CD20. These immunological markers are fundamental in the identification of mantle cell lymphoma from other types of lymphoma (20). Moreover, cytogenetic analysis of MCL shows a rearrangement and translocation t (11,14) on chromosome 11, accompanied by overexpression of the cyclin D1 antigen (22). The cornerstone of adult intussusception treatment is surgery. Laparoscopy can also assist in the preoperative diagnosis of intussusception in cases where the diagnosis is suspected (23). the surgical resection depends on the location and extent of the lesion, and can range from a localized bowel resection to an hemicolectomy (12). In our case, the patient underwent a right hemicolectomy. Thanks to the latest combinations of monoclonal antibodies and chemotherapy, MCL response rates have improved to reach 80–95 % (24). The R-CHOP (rituximab- cyclophosphamide, doxorubicin, vincristine, and prednisone) regimen has been chosen as a gold standard for treatment of mantle cell lymphoma. (20,23) In our case, we opted for eight cycles of chemotherapy involving rituximab-CHOP.

The extent of gastrointestinal invasion seems to have had an impact on the treatment strategy. Indeed, patients with a single lesion were more likely to be treated with surgery, whereas patients with multiple lesions (involving more than two organs) were more often treated with intensive systemic chemotherapy (25).

Over the last few years, stem cell transplantation has been the only treatment that guarantees prolonged remissions, especially in young patients. However, it has always been limited by toxicity, age-related restrictions and the availability of this procedure in some country which was the case for our patient.

In summary, we reported the case of 34-year-old patient complaining of abdominal pain due to an ileocolic intussusception secondary to mantle cell lymphoma suspected on CT scan and which was successfully treated with surgical resection followed by chemotherapy.

Our case highlights that early diagnosis and a timely indication of surgical treatment, combined with chemotherapy, are crucial for favorable outcomes.

## 4 Conclusion

Mantle cell lymphoma is a rare form of non-Hodgkin's lymphoma with non-specific gastrointestinal manifestations. Symptoms appear at an advanced stage of the disease or in a complicated form.

Ileocolic intussusception is an extremely rare manifestation of this disease.

Surgery combined with chemotherapy is the mainstay of treatment that has significantly improved the prognosis of this disease.

The definitive diagnosis is confirmed by histological analysis of the surgical specimen.

## Patient consent

Written informed consent was obtained from the patient for publication of this case.

report and accompanying images. A copy of the written consent is available for review by the Editor-in-Chief of this journal on request.

## Provenance and peer review

Not commissioned, externally peer-reviewed.

## Ethical approval

Not required.

## Funding

This research did not receive any specific grant from funding agencies in the public, commercial, or not-for-profit sectors.

## Author contributions

Mohamed Amine Tormane and Ghazi Laamiri did the conception and design of the work, the data collection, and the data analysis and interpretation.

Hazem Beji and Houda Gazzeh: did the critical revision of the article.

Mahdi Bouassida, and Hassen Touinsi: did the final approval of the version to be published.

All authors read and approved the final manuscript.

## Guarantor

Mohamed Amine Tormane.

Ghazi Laamiri.

## Declaration of competing interest

No conflicts of interest.
